# Clinical Cases of Tick-Borne Diseases in Dogs During the Autumn-Winter Season in Poland

**DOI:** 10.3390/pathogens13121132

**Published:** 2024-12-21

**Authors:** Ismena Gałęcka, Zhuowei Ma, Xuenan Xuan, Remigiusz Gałęcki

**Affiliations:** 1 Department of Epizootiology, Faculty of Veterinary Medicine, University of Warmia and Mazury in Olsztyn, 10-719 Olsztyn, Poland; 2 National Research Center for Protozoan Diseases, Obihiro University of Agriculture and Veterinary Medicine, Obihiro 080-8555, Japan; 3 Department of Veterinary Prevention and Feed Hygiene, Faculty of Veterinary Medicine, University of Warmia and Mazury in Olsztyn, 10-719 Olsztyn, Poland

**Keywords:** *Ixodes ricinus*, *Dermacentor reticulatus*, *Borrelia* spp., *Babesia* spp., *Mycoplasma* spp., *Anaplasma* spp.

## Abstract

Tick-borne diseases (TBDs) pose a growing threat to companion animals, especially dogs, due to the increasing abundance of tick populations in Europe, driven by climate change, urbanization, and the mobility of humans and animals. This study aimed to assess the prevalence of tick-borne pathogens in clinically ill dogs suspected of having developed TBDs during the autumn-winter season, as well as to detect pathogens in ticks collected during the same period in the Warmian–Masurian Voivodeship in Poland. A total of 30 dogs with clinical symptoms of babesiosis and 45 ticks from dogs were acquired for this study. Clinical symptoms in dogs included elevated body temperature > 39.0 °C (73.3%), anemia (56.7%), thrombocytopenia (80%), and dark urine (53.3%). Co-infections with *Babesia* spp. were identified in two combinations (*Babesia* spp. and *Mycoplasma* spp. (*n* = 5), *Babesia* spp. and *Borrelia* spp. (*n* = 2)) and one co-infection with *Anaplasma* spp. and *Borrelia* spp., highlighting the complexity of TBD diagnosis and treatment. The analyzed tick species were *Ixodes ricinus* (86.7%; *n* = 39; 18 females and 21 males) and *Dermacentor reticulatus* (13.3%; *n* = 6; 4 females and 2 males). In *I. ricinus*, *Babesia* spp. were identified in 7.7% (3/39), *Mycoplasma* spp. in 7.7% (3/39), *Borrelia* in 25.6% (10/39), and *Anaplasma* spp. in 10.3% (4/39). In *D***.** *reticulatus*, only two pathogens—*Borrelia* spp. and *Anaplasma* spp.—were detected, both only once (16.7%; 1/6). No significant differences were observed between the prevalence of the studied pathogens and tick species, sex, or developmental stage. This study emphasizes the year-round risk of TBDs in dogs, particularly during the autumn-winter months, and underscores the need for continuous vigilance in tick prevention, broad-spectrum diagnostics, and treatment strategies.

## 1. Introduction

Tick-borne diseases (TBDs) represent a major concern in veterinary medicine due to their significant impact on the health and welfare of companion animals. These diseases are transmitted by various species of ticks and can lead to a wide range of clinical manifestations, from mild symptoms to severe, life-threatening conditions [1]. The importance of TBDs in companion animals has increased due to the growing abundance and geographic range of ticks, driven by factors such as climate change, urbanization, and increased mobility of both humans and animals [2,3]. Ticks act as vectors for numerous pathogens, including bacteria, viruses, and protozoa, which can infect companion animals [4,5]. The interactions between these vectors and pathogens are complex and influenced by environmental conditions, host availability, and ecological dynamics [5]. The spread of tick populations into new areas, often facilitated by climate change, has led to the emergence and re-emergence of tick-borne diseases in regions previously considered non-endemic [6,7,8]. This geographic expansion poses new challenges for veterinary practitioners, as they must now manage diseases that were once rare or unknown in their regions [7]. Companion animals, particularly dogs and cats, can serve as indicators of TBDs by providing early warning signs of emerging threats to human health [9]. Moreover, the close relationship between companion animals and their owners increases the risk of zoonotic transmission, highlighting the importance of integrated approaches to disease management that encompass both veterinary and human health perspectives [10]. These links are also important in the context of the “One Health” initiative.

Veterinary medicine has responded to the growing threat of TBDs through advances in diagnostic methods, treatment protocols, and preventive measures [11,12]. Early and accurate diagnosis is crucial for effective management of these infections, as many TBDs can present with non-specific clinical signs that overlap with other conditions [13,14]. Molecular diagnostic techniques, such as the polymerase chain reaction (PCR), facilitate the detection and identification of tick-borne pathogens, thus contributing to more accurate treatment and management strategies [15]. Preventive measures, including tick control and vaccination, play a vital role in reducing the incidence of TBDs in companion animals [4,16,17,18,19]. Tick-borne diseases have a substantial economic impact on veterinary practices and pet owners. Treatment often requires prolonged medical care, including hospitalization, medication, and follow-up visits, which generates significant financial costs [20].

In Europe, the prevalence of TBDs in companion animals, particularly dogs, has risen significantly in recent decades. The most prominent TBDs affecting dogs in Europe are babesiosis, mycoplasmosis, borreliosis (Lyme disease), and anaplasmosis, each posing distinct challenges for veterinary medicine [21,22,23].

Babesiosis is caused by protozoan parasites of the genus *Babesia*, and it is transmitted primarily by *Dermacentor* spp., *Ixodes* spp., and *Rhipicephalus* spp. [24]. In Europe, *Babesia canis* is most commonly implicated in canine babesiosis (CB) and is mainly transmitted by *D. reticulatus* ticks. In southern parts, CB is caused by *B. vogeli*, which is mostly transmitted by *R. sanguineus* [25]. It leads to hemolytic anemia, fever, and splenomegaly in infected dogs [21]. The disease is particularly prevalent in Southern and Eastern Europe [21]; however, the incidence of babesiosis has also increased in France, Italy, and Germany [9,26].

Canine mycoplasmosis is caused by *Mycoplasma haemocanis*, yet another emerging tick-borne pathogen, and *R. sanguineus* is suspected to be its main vector. This bacterium infects red blood cells, leading to hemolytic anemia, particularly in immunocompromised or splenectomized dogs [27,28]. The prevalence of canine mycoplasmosis has been reported across European countries, including Spain, Italy, and Greece [9,20]. The spread of *R*. *sanguineus* in temperate climates has contributed to the broader distribution of mycoplasmosis in Europe [20,29].

Borreliosis, also known as Lyme disease, is caused by the spirochete *Borrelia* spp. and is transmitted primarily by *I. ricinus* ticks in Europe [24]. Lyme disease presents a wide range of clinical manifestations in dogs, including lameness, fever, and renal complications [15]. The higher prevalence of Lyme disease is observed in Central and Northern Europe, where *I*. *ricinus* is most prevalent [30]. Countries such as Germany, Switzerland, and the Czech Republic have reported significant seroprevalence rates in dogs, which testifies to the widespread presence of the tick vector and the pathogen [31].

Anaplasmosis, caused by the bacterium *Anaplasma phagocytophilum*, is also an important TBD in dogs. This disease leads to fever, lethargy, joint pain, and thrombocytopenia in infected animals [32,33,34]. Seroprevalence studies have reported high infection rates in dogs from various countries, including Sweden, Norway, and the Netherlands [33].

The increasing prevalence of these TBDs among companion animals in Europe underscores the need for vigilant surveillance, effective tick control measures, and continued research into diagnostic and therapeutic approaches [8,9,35,36]. Climate change and human activities continue to influence tick ecology and distribution, and the risk of these diseases is likely to grow and pose ongoing challenges for veterinary practitioners and pet owners [37].

The co-occurrence of multiple TBDs in dogs poses a significant challenge in veterinary medicine, affecting therapeutic success, and potentially leading to severe complications or death [38,39,40,41]. Dogs can be simultaneously infected with several tick-borne pathogens belonging to the genera *Babesia*, *Mycoplasma*, *Borrelia*, and *Anaplasma* [9]. Co-infections complicate diagnosis and treatment due to overlapping clinical symptoms and interactions between the pathogens. For instance, Carrade et al. [34] reported that dogs infected with both *B. burgdorferi* and *A. phagocytophilum* exhibited more severe clinical signs than those infected with one pathogen only. Additionally, co-infections may increase the risk of treatment failure and recurrence of clinical symptoms because the immune response to one pathogen may be compromised by the presence of another pathogen [20,34].

Over time, the distribution and seasonality of tick populations have shifted, leading to changes in the prevalence and phenology of TBDs [9]. Climate change and human activity have expanded the habitats suitable for ticks, resulting in a broader geographic distribution and increased tick activity during traditionally low-risk periods, such as winter [24,29]. Ticks, in particular *I. ricinus*, have adapted to milder winter temperatures, which has enabled them to remain active and capable of transmitting diseases year-round [24,29,42]. The studies by Probst et al. documented active tick infestations in dogs and cats in winter months, which highlights the need for year-round vigilance in tick prevention and monitoring [24,43]. In Central Europe, *I. ricinus* typically exhibits a bimodal pattern of questing activity, with a prominent peak in spring and a smaller, sometimes absent, peak in autumn. During the summer months, higher temperatures and lower relative humidity suppress their activity [29,44,45,46]. Similarly, *D. reticulatus*, another important vector species, shows bimodal activity peaks. However, its spring activity begins earlier and concludes before high summer temperatures, while its autumn activity starts later and extends well into cooler months [45,47]. Notably, reports from Poland document the activity of *D. reticulatus* even during winter months [48,49], further emphasizing the variability in tick questing behavior across seasons.

The prevalence of TBDs in the last and first months of the year has not been extensively investigated, and our understanding of the seasonal dynamics of these diseases is incomplete [43]. Despite the fact that TBDs’ impact on companion animals is increasingly recognized, several research gaps remain. Above all, little is known about the seasonal dynamics and year-round activity of tick populations, particularly during winter months, which affect the prevalence of TBDs [42,43]. Additionally, the interactions and clinical implications of co-infections in dogs have not been fully elucidated, which complicates diagnosis and treatment strategies [34]. Finally, the prevalence of TBDs should also be investigated in the last months of the year to better understand and mitigate the risks associated with variations in seasonal tick activity [43]. Therefore, the aim of this study was to identify pathogens in clinically ill dogs suspected of developing TBDs during the autumn-winter season. Ticks were collected in a veterinary clinic during the same period, and the pathogens infecting ticks were identified with the use of molecular techniques. The study hypothesizes that there is a significant risk of TBDs in dogs throughout the year.

## 2. Materials and Methods

### 2.1. Sample Collection

In 2021, 2022, and 2023, samples were collected between November and February from dogs that were patients at the Veterinary Polyclinic of the Faculty of Veterinary Medicine at the University of Warmia and Mazury in Olsztyn. The material was collected by veterinarians as part of standard clinical procedures that did not require the consent of the ethics committee. The veterinarians obtained verbal consent from the animals’ owners to use the material in scientific research. Blood was sampled from animals that were suspected of babesiosis and had ticks removed from their bodies in the previous two months. A total of 30 dogs were included in the study based on the results of clinical and diagnostic tests.

The following data were acquired from dogs suspected of babesiosis: sex (male, female), breed (purebred, mixed breed), body weight (up to 5 kg—small; from 5 kg to 25 kg—medium; over 25 kg—large), coat (short-haired, wire-haired, long-haired), and protection against ectoparasites (absent, present). The animals were also assessed for the following clinical symptoms: fever, anemia, thrombocytopenia, and dark urine (absent, present). Regarding the dogs’ utility, two patients occasionally participate in hunting. Despite this, all of them should be described as pet dogs. The interviews revealed that all dogs included in the study came from the Warmian–Masurian Voivodeship and had not traveled outside the region in the previous two years. The mentioned region is characterized by its abundant lakes and forested areas, which provide a conducive environment for ticks. According to the Polish Institute of Meteorology and Water Management (IMGW), the autumn and winter seasons of 2021–2023 recorded mild temperatures with above-average rainfall and high relative humidity, creating favorable conditions for tick survival and activity. According to the National Institute of Public Health (NIH), this region of Poland is endemic for Lyme Disease and TBE (https://wwwold.pzh.gov.pl/oldpage/epimeld/index_a.html; accessed on 10 December 2024).

In addition, 45 ticks were collected during veterinary procedures or through the involvement of animal owners, who removed ticks from their dogs either after outdoor activities or directly from the animals’ skin. Ticks were submitted by owners in exchange for a diagnostic analysis of pathogen presence, allowing us to gather samples opportunistically. The ticks were collected not only from dogs with clinical signs of TBD but also from uninfected individuals. Due to the variability in tick collection sources and timing, tick data were used as supplementary information to complement diagnostic evaluations for TBDs in the studied period. Due to the character of tick collection, we were not able to establish the number of dogs from which ticks were obtained. The sampled ticks were transported to the Biological Hazard Laboratory at the Faculty of Veterinary Medicine of the University of Warmia and Mazury in Olsztyn. Tick species, sex, and developmental stages were identified based on their morphological characteristics under the Leica M165C stereoscopic microscope (Leica, Wetzlar, Germany) using an existing taxonomic key [50].

### 2.2. Blood Morphological and Biochemical Analyses

Blood was collected by puncturing the *vena cephalica* into a tube containing a coagulation activator for biochemical analyses and EDTA for morphological analyses immediately after obtaining the sample. The samples were left to stand at room temperature for at least 30 min. To obtain the serum, the collected material was centrifuged at room temperature for 10 min at 3000 rpm.

In the biochemical analysis, the concentrations of alkaline phosphatase (ALKP), alanine aminotransferase (ALT), aspartate aminotransferase (AST), blood urea nitrogen (BUN), creatinine (CREA), and total bilirubin (TBIL) were determined using the Idexx Catalyst One Chemistry Analyzer (IDEXX Laboratories Inc., Westbrook, CA, USA), according to the manufacturer’s instructions. The morphological analysis was performed using the ProCyte Dx Hematology Analyzer (IDEXX Laboratories Inc., Westbrook, CA, USA).

### 2.3. Identification of Pathogens Under Clinical Conditions

To identify *Babesia* spp., blood smears were prepared according to standard veterinary protocols. A small drop of fresh blood was placed on one end of a clean glass microscope slide. Using a second slide held at a 30–45 degree angle, the drop was swiftly spread across the surface to create a thin smear. This technique was repeated to produce three smears for a single patient to ensure accuracy and provide multiple samples for examination [21].

The prepared smears were stained using the Diff-Quick set method, which is a modification of the Romanowsky stain technique. The staining process involved three steps. First, the slide was fixed in methanol for around 1–2 min. Then, it was submerged in a solution of eosinophilic stain (Solution I (Merck, Darmstadt, Germany)) for approximately 5–10 s and dipped in a basophilic stain (Solution II (Merck, Darmstadt, Germany)) for another 5–10 s. After each dip, the slide was briefly rinsed with distilled water to remove excess stain. The stained slides were air-dried completely before microscopic examination. The initial examination was conducted at low magnification (10× objective) to assess the quality of the smear and to identify areas where red blood cells (RBCs) were evenly distributed without overlapping. The final examination was performed under an oil immersion objective lens at 100× magnification to observe the fine details of any organisms present inside or outside blood cells. An optical microscope ECLIPSE Si (Nikon, Tokyo, Japan) was used for the research.

In the microscopic analysis, *Babesia* spp. were identified based on the distinctive morphological features of merozoites inside RBCs. Typically, they appear as small, round, or pear-shaped organisms that often occur in pairs or tetrads and can be distinguished based on their size and intracellular location [21].

The smears were also examined for the presence of other vector-borne pathogens, such as *Anaplasma* spp. and *Mycoplasma* spp. *Anaplasma* spp. appear as small, dark-staining inclusions (intracytoplasmic morulae) at the periphery of white blood cells (granulocytes), whereas *Mycoplasma* spp. are seen as small, coccoid forms on the surface of RBCs [32,33,51].

### 2.4. DNA Extraction

Blood samples were removed from storage, left to stand at room temperature for 15 min, and then carefully transferred into sterile test tubes. Genomic DNA was isolated from each sample using the Genomic Mini AX Blood kit (A&A Biotechnology, Gdynia, Poland), according to the manufacturer’s instructions. Ticks were removed from test tubes, left to air-dry at room temperature for 15 min, and individually crushed with a sterile glass rod in sterile test tubes. Genomic DNA was extracted from each tick using the Genomic Mini AX Tissue kit (A&A Biotechnology, Gdynia, Poland), according to the manufacturer’s instructions. For both types of samples, DNA was eluted in 40 µL of TE buffer. The concentration of the final DNA product was measured with the Nano Drop 2000 spectrophotometer (Thermo Fisher Scientific, Waltham, MA, USA). The extracted DNA was stored at −20 °C until further examination.

### 2.5. Polymerase Chain Reaction

Primer sequences and PCR conditions are detailed in Table 1. Each reaction was performed in a total volume of 25 µL, comprising 2.5 µL of 10 × Standard Taq Reaction Buffer (Biolabs, Boston, MA, USA), 0.5 μL of 10 mM dNTPs (Biolabs, Boston, MA, USA), 0.5 μL of 10 μM solution of each primer, 1 μL of extracted DNA, 0.125 µL of Taq DNA polymerase (Biolabs, Boston, MA, USA), and 19.875 µL of double-distilled water. The reactions were run on the Veriti Thermal Cycler (Applied Biosystems, Foster City, CA, USA). In the negative control sample, DNA was replaced with double-distilled water. The positive control sample contained DNA from each analyzed pathogen. The PCR products were separated on 1.5–2.5% agarose gel with a 100 bp DNA ladder as the molecular weight size marker, stained with ethidium bromide, and visualized under a UV transilluminator.

### 2.6. Sequencing

In total, 8 samples positive for *Babesia* spp., 4 samples positive for *Mycoplasma* spp., 3 samples positive for *Borrelia* spp., and 1 sample positive for *Anaplasma* spp., collected from dogs, were subjected to DNA sequencing. The samples were selected randomly using a random number generator. Cycle-sequencing reactions were conducted with the use of the described primers, the BigDye Terminator Cycle Sequencing Kit (Applied Biosystems, Foster City, CA, USA), and the ABI PRISM 3100 Genetic Analyzer (Applied Biosystems, Foster City, CA, USA). The resulting nucleotide sequences were edited in BioEdit and compared against GenBank data with the BLAST-NCBI tool and uploaded to GenBank under following accession numbers: PQ108647, PQ108648, PQ108649, PQ108686, PQ108687, PQ108688, PQ108689 and PQ108690 for *Babesia* spp., PQ177842, PQ192186 and PQ192248 for *Mycoplasma* spp., PQ177472 for *Anaplasma* spp. and PQ206404, PQ206405 and PQ206406 for *Borrelia* spp. The phylogenetic analyses of the obtained sequences and the corresponding GenBank sequences were performed by the neighbor-joining (NJ) method in MEGA 10.1.17. Bootstrap confidence values were calculated in 10,000 replicates to estimate branching reliability.

### 2.7. Statistical Analysis

The Fisher’s exact test was used to determine the presence of significant differences between dog breed, age, sex, size, coat type, preventive treatment, and the prevalence of *Anaplasma* spp., *Mycoplasma* spp., and *Borrelia* spp. The Fisher’s exact test was also applied to determine significant differences between tick species and sex, and the prevalence of the studied pathogens. Mean values (m), standard deviation (SD), and standard error (SE) were calculated for the results of morphological and biochemical blood tests. For the prevalence of selected clinical symptoms and pathogen detection, 95% CI was calculated. Confidence intervals for morphological and biochemical blood parameters and patient characteristics have been placed in Supplementary Material S1. Statistical significance was set at *p* < 0.05. Data were processed in the Statistica 13.3 program (TIBCO Software Inc., Palo Alto, Santa Clara, SC, USA).

## 3. Results

### 3.1. Clinical Examination

The study was conducted on 30 dogs with various characteristics (Table 2). Of these, 11 dogs (37%) were purebred and 19 dogs (63%) were mixed breeds. The study group consisted of 13 males (43%) and 17 females (57%). The age range was as follows: five dogs (16.7%) were ≤1-year-old, 20 dogs (66.7%) were between 2 and 7 years, and five dogs (16.7%) were ≥8-years-old. In terms of size, five dogs (16.7%) were small breeds, 14 dogs (46.7%) were medium breeds, and 11 dogs (36.7%) were large breeds. In terms of coat type, 11 dogs were short coated (37%), 16 dogs were long coated (53%), and three dogs were wire coated (10%). Six dogs (20%) were protected against ectoparasites (ticks), while 24 dogs (80%) were not.

Body temperature was elevated (>39.0 °C) in 22 dogs (73.3%; CI: 57–89.6%.) (m = 39.75 °C, SD = 0.58, SE = 0.12), while eight dogs (26.7%; CI: 10.4–43%.) had normal body temperature (≤39.0 °C) (m = 38.45 °C, SD = 0.47, SE = 0.17). Seventeen dogs were diagnosed with anemia (56.7%; CI: 38.9–74.5%) and red blood cell levels (m = 4.04 M/µL, SD = 1.33, SE = 0.32), whereas 13 dogs (43.3%; CI: 25.5–61.1%) had normal red blood cell levels (m = 6.55 M/µL, SD = 0.79, SE = 0.22). Based on leukocyte counts, leukocytosis was observed in nine dogs (30%; CI: 12.5–47.5%) (m = 20.07 K/µL, SD = 1.87, SE = 0.62) and leukopenia was observed in eight dogs (26.7%; CI: 10.4–43%) (m = 4.11 K/µL, SD = 0.71, SE = 0.25), while 13 dogs (43.3%; 25.5–61.1%) had normal leukocyte counts (m = 9.65 K/µL, (SD = 3.32, SE = 0.92). Low platelet counts (thrombocytopenia) were observed in 24 dogs (80%; CI: 67.2–92.8%.) (m = 75.33 K/µL, SD = 33.77, SE = 6.89), whereas six dogs (20%; CI: 4–36%) had normal platelet counts (m = 162.83 K/µL, SD = 50.21, SE = 20.49). Dark urine was present in 16 dogs (53.3%; CI: 35.5–71.1%) and absent in 14 dogs (46.7%; CI: 28.9–64.5%). Creatinine levels were elevated (>1.8 mg/dL) in nine dogs (30%; CI: 13.2–46.8%.) (m = 111.36 mg/dL, SD = 184.34, SE = 61.45) and normal in 21 dogs (70%; CI: 53.2–86.8%) (m = 1.53 mg/dL, SD = 0.64, SE = 0.14). Blood urea nitrogen levels were pathological (>27 mg/dL) in 10 dogs (33.3%; CI: 30.5–69.5%) (m = 192.67 mg/dL, SD = 155.04, SE = 53.01) and normal in 20 dogs (66.7%; CI: 53.3–86.7%) (m = 36.85 mg/dL, SD = 36.02, SE = 8.06). Aspartate aminotransferase (AST) levels exceeded the norm (>50 U/I) in 19 dogs (63.3%) (m = 184.36 U/I (SD = 207.91, SE = 47.71) and were normal in 11 dogs (36.7%; CI: 36.7–63.3%) (m = 33.82 U/I, SD = 14.77, SE = 4.45). Alanine aminotransferase (ALT) levels were pathological (>125 U/I) in six dogs (20%; CI: 4–36%) (m = 603.67 U/I (SD = 453.97, SE = 185.34) and normal in 24 dogs (80%; CI: 67.2–92.8%) (m = 96.27 U/I, SD = 56.47, SE = 11.53). Alkaline phosphatase (ALKP) levels were elevated (>212 U/I) in seven dogs (23.3%; CI: 11.7–41.7%) (m = 1078.43 U/I (SD = 478.41, SE = 180.78) and normal in 23 dogs (76.7%; CI: 72.3–89.7%) (m = 180.91 U/I, SD = 264.68, SE = 55.19). Elevated bilirubin levels (*icterus*) were observed in four dogs (13.3%; CI: 1.2–22.8%) (m = 2.8 mg/dL, SD = 1.26, SE = 0.63), whereas 26 dogs (86.7%; CI: 76.7–92.3%) had normal bilirubin levels (m = 0.41 mg/dL, SD = 0.24, SE = 0.05). Twenty-nine of the thirty dogs treated for babesiosis received imidocarb and 2 of them died. One dog was treated with doxycycline and also died. In this case, imidocarb had been previously administered twice, but it had no therapeutic effect, and the patient was switched to doxycycline. The overall mortality rate was 10% (CI: 1.2–21.8%). The remaining 27 dogs (90%; CI: 83–97%) treated with imidocarb recovered. Symptomatic treatment was also administered, depending on the symptoms (antipyretic, nephro- and hepatoprotective, fluid therapy) and the patient’s health condition.

### 3.2. Polymerase Chain Reaction Results

Out of the 30 cases diagnosed using standard clinical methods, 29 cases (96.7%) were confirmed by PCR. The adopted methodological approach did not yield a positive result in only one case (case no. 26—mixed breed, 6 years old, female, 28 kg, long coat, Table 3).

In this dog, PCR test results confirmed co-infection with *Anaplasma* spp. and *Borrelia* spp. This was the only case confirming *Anaplasma* spp., while *Borrelia* spp. were found in three out of thirty dogs (10%), including two purebred dogs and one mixed-breed dog.

*Mycoplasma* spp. was identified in five of thirty dogs (16.7%). *Mycoplasma* spp. was identified only in mixed-breed dogs (26.3% of the mixed-breed group), while no purebred dogs tested positive for these pathogens. The results of polymerase chain reactions are presented in Table 3. The co-infection was detected in eight dogs *(Babesia* spp. and *Mycoplasma* spp. (*n* = 5), *Babesia* spp. and *Borrelia* spp. (*n* = 2), *Anaplasma* spp. and *Borrelia* spp. (*n* = 1); Table 3).

However, the Fisher’s exact test revealed no significant differences between the prevalence of *Anaplasma* spp., *Mycoplasma* spp., or *Borrelia* spp. and the studied dogs’ breed (*p* > 0.05), age (*p* > 0.05), sex (*p* > 0.05), size (*p* > 0.05), coat type (*p* > 0.05), or preventive treatment (*p* > 0.05).

### 3.3. Sequencing

Based on Genbank database, out of eight *Babesia*-positive sequenced samples, seven were identified as *B. canis* and one as *B. vogeli*. Our sequences, PQ108649 and PQ108690, were identical to *B. canis* previously detected in Romania from a golden jackal, a European wildcat, and a dog (Acc. No. KX712122, MW939359, HQ662634, respectively). Two other samples (PQ108647 and PQ108648) showed very high similarity (99.93%) to the same sequences. Sequence PQ108687 showed 99.93% similarity to *B. canis* obtained from a European wolf from Croatia (KY359360), horses from Italy (KX839230, KX839231), and a dog from Croatia. Sequences PQ108688 and PQ108689 also showed very high similarity (99.86%) to the *B. canis* detected in a donkey from Turkey (MG569903), a golden jackal from Serbia (KY747491), and a European wolf from Croatia (KY359360). Our sequence PQ108686 was identical to *B. vogeli* collected from dogs in Romania (HQ662635) and Egypt (AY371197).

The sequence of the *Anaplasma*-positive sample from our study (PQ177472) was identified as uncultured *Anaplasma* sp. and was identical to those obtained from *I. ricinus* from Estonia (HQ629922), sheep in Norway (CP015376) and dogs in Japan, Croatia, Austria, Germany, and Poland (LC334014, KY114936, JX173651, JX173652, DQ105667).

Regarding *Mycoplasma*, three (PQ177842, PQ192186, PQ192248) out of four sequenced samples were recognized as *M. haemocanis* and were identical to those obtained from dogs in Portugal, Switzerland, Turkey, and Italy (GQ129118, GQ129119, EF416567, MG594502, respectively). The other sample was identified as *Candidatus* M. haematoparvum and showed 100% similarity to sequences recovered from dogs from Italy, Switzerland, and Cuba (MH094850, GQ129112, EF416569, MZ221181) as well as from a human from the USA (KF366443).

Sequencing of *Borrelia*-positive samples revealed the presence of two genospecies. Two of our samples were identified as *B. garinii*. One (PQ206404) was identical and the other one (PQ206406) showed 99.58% similarity to the isolates recovered from *I. ricinus* in France and Germany (KY273110, MW489079). The third sample (PQ206405) was identical to *B. burgdorferi* detected in a Eurasian badger from Poland (OP559187) and *I. ricinus* from the Czech Republic and Great Britain (AF497979, OL848352).

**Figure 1:**
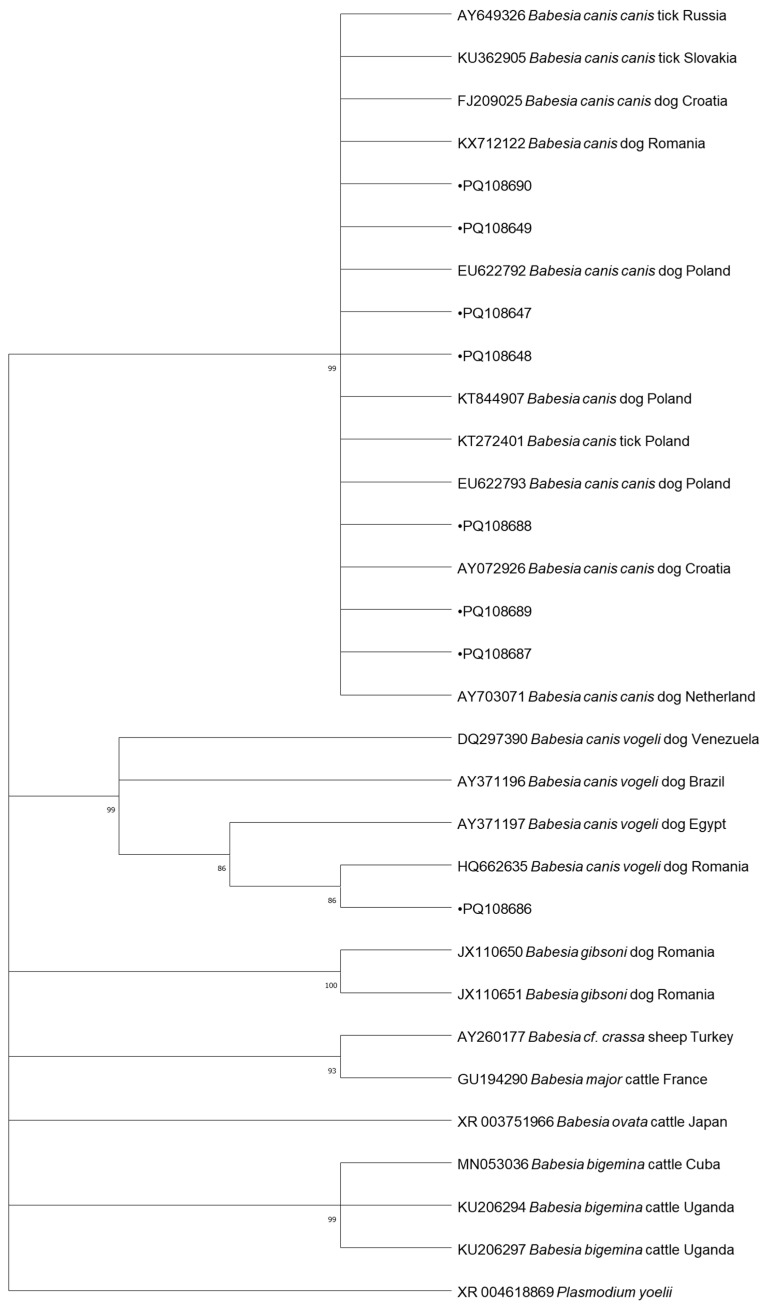
Phylogenetic topology for the neighbor-joining analysis of the 18S ribosomal RNA gene, partial sequence of *Babesia* spp. The unique haplotypes identified in this study are labeled with the corresponding sequence identification numbers and with dots. GenBank reference sequences are indicated in the tree. Bootstrap confidence values were calculated in 10,000 replicates to estimate branching reliability.

**Figure 2:**
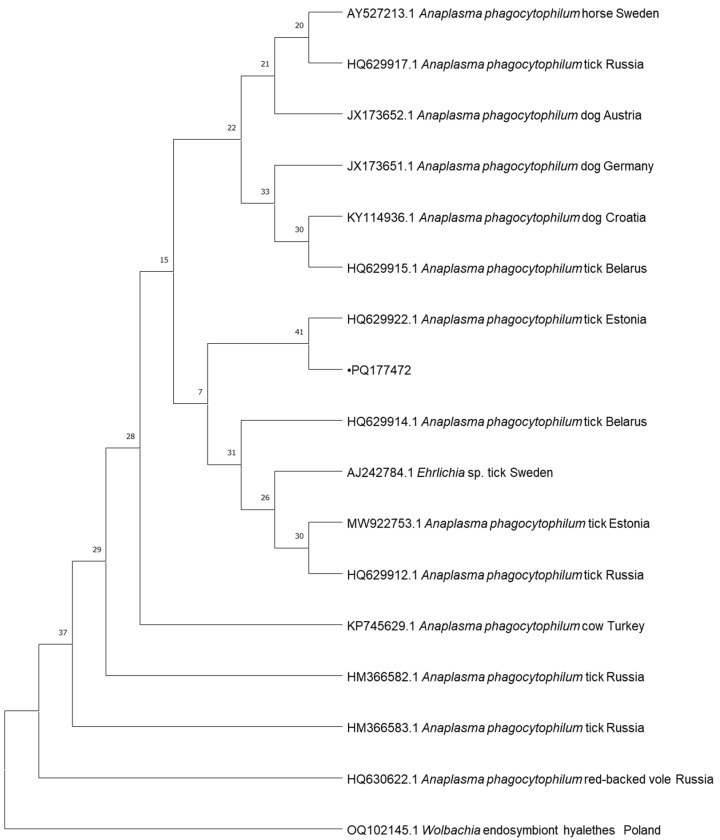
Phylogenetic topology for the neighbor-joining analysis of the 18S ribosomal RNA gene, partial sequence of *Anaplasma* spp. The unique haplotypes identified in this study are labeled with the corresponding sequence identification numbers and with dots. GenBank reference sequences are indicated in the tree. Bootstrap confidence values were calculated in 10,000 replicates to estimate branching reliability.

**Figure 3:**
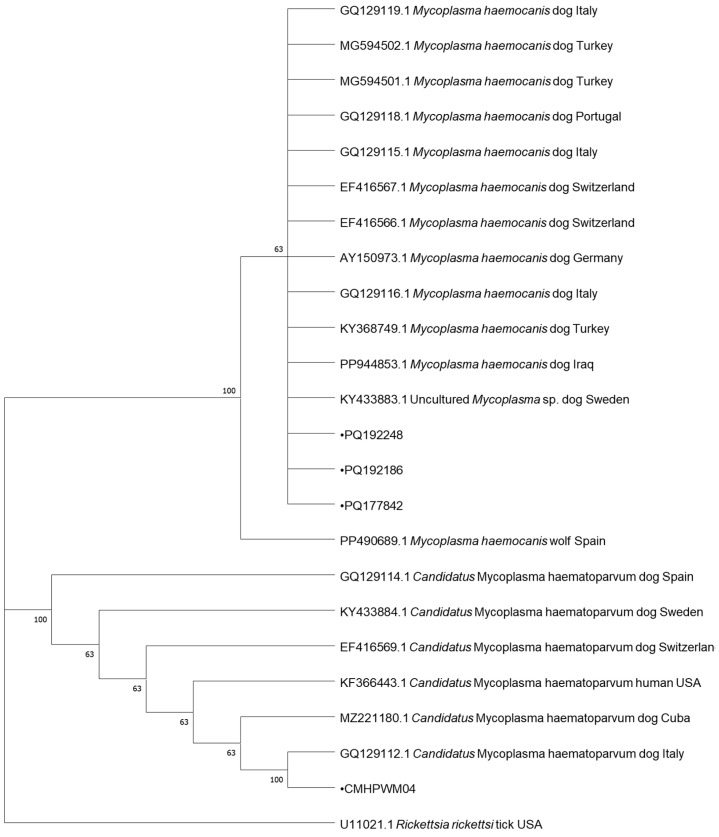
Phylogenetic topology for the neighbor-joining analysis of the 18S ribosomal RNA gene, partial sequence of *Mycoplasma* spp. The unique haplotypes identified in this study are labeled with the corresponding sequence identification numbers and with dots. GenBank reference sequences are indicated in the tree. Bootstrap confidence values were calculated in 10,000 replicates to estimate branching reliability.

**Figure 4:**
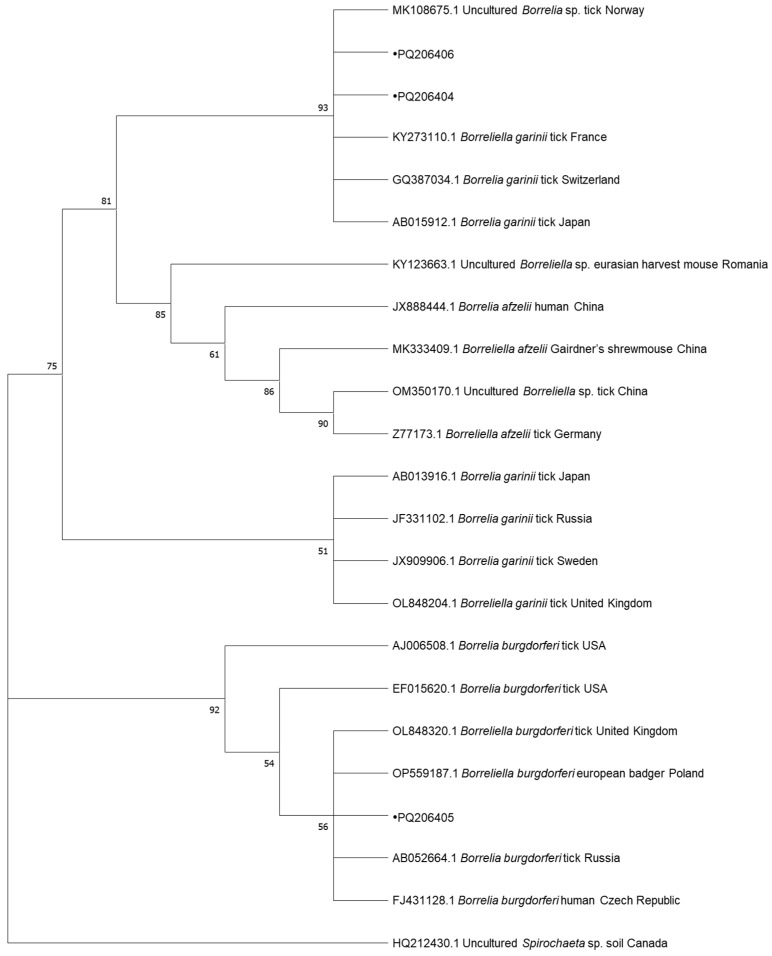
Phylogenetic topology for the neighbor-joining analysis of the 18S ribosomal RNA gene, partial sequence of *Borrelia* spp. The unique haplotypes identified in this study are labeled with the corresponding sequence identification numbers and with dots. GenBank reference sequences are indicated in the tree. Bootstrap confidence values were calculated in 10,000 replicates to estimate branching reliability.

The phylogenetic trees for the neighbor-joining analysis of the obtained sequences are presented in Figure 1, Figure 2, Figure 3 and Figure 4.

### 3.4. Ticks

The sample consisted of 45 adult ticks belonging to two species: *I. ricinus* (86.7%; *n* = 39 and *D. reticulatus* (13.3%; *n* = 6). The sample was nearly balanced in terms of sex distribution, with 22 females (48.9%) and 23 males (51.1%). *Babesia* spp. were detected in 6.7% (*n* = 3; 95% CI: 0–10.6%) of the analyzed ticks, *Mycoplasma* spp. in 6.7% (*n* = 3; 95% CI: 0–10.6%), *Borrelia* spp. in 24.4% (*n* = 11; 95% CI: 14.4–36.6%), and *Anaplasma* spp. in 11.1% (*n* = 5; 95% CI: 2.9–17.1%). The prevalence of the studied pathogens varied between tick species. In *I. ricinus*, *Borrelia* spp. was dominant (*n* = 10; 25.6%; 95% CI: 14.3–37.7%), followed by *Babesia* spp. (*n* = 3; 7.7%; 95% CI: 0.3–12.9%), *Anaplasma* spp. (*n* = 4; 10.3%; 95% CI: 2.6–15.4%), and *Mycoplasma* spp. (*n* = 3; 7.7%; 95% CI: 0.3–12.9%). In contrast, *D. reticulatus* did not harbor *Babesia* spp. or *Mycoplasma* spp., but *Borrelia* spp. and *Anaplasma* spp. were detected only in one specimen each (*n* = 1; 95% CI: 0–46.5%). The detailed results are presented in Table 4. No significant differences were found between tick species (*p* > 0.05) or sex (*p* > 0.05) and the prevalence of selected pathogens. Co-infections were detected only in three ticks of the species *I. ricinus.* Co-infection with *Anaplasma* spp. and *Borrelia* spp. was detected in one female, while co-infection with *Anaplasma* spp., *Borrelia* spp., and *Mycoplasma* spp. was detected in one male and *Babesia* spp. and *Borrelia* spp. in another male.

## 4. Discussion

The study confirmed that TBDs pose a significant threat to canine health even during colder months, which challenges the conventional understanding that tick activity decreases in autumn and winter. Recent studies have shown that climate change is contributing to the extended activity of tick populations across seasons [6,56,57]. Tick species such as *I. ricinus* and *D. reticulatus* have adapted to milder winter temperatures, allowing them to transmit pathogens during periods that are generally considered low-risk [29,43]. The extended activity of ticks during colder months, as highlighted in the present study, aligns with findings from various European regions. For instance, research conducted in Poland documented significant winter activity of *D. reticulatus* in Lower Silesia, a phenomenon attributed to local climatic conditions and the species’ adaptability to cooler environments [49]. Similarly, Buczek et al. observed that fluctuations in weather factors, such as temperature and humidity, directly influence the questing behavior of adult *D. reticulatus* in eastern Poland [48]. In Hungary, Hornok reported allochronic seasonal peaks for *Dermacentor* spp., with activity extending into colder months, further demonstrating the resilience of this species under a continental climate [45]. Extended activity has been documented in Northern Europe, where Kjellander et al. observed active tick infestations on roe deer during winter in Sweden [39].

The influence of climate change on tick behavior cannot be overlooked, as milder winter temperatures across Europe are fostering prolonged activity periods for multiple tick species, including *I. ricinus* and *D. reticulatus* [29]. Studies in eastern Austria also highlight that seasonal dynamics are significantly impacted by acaricides and repellents, though their efficacy in winter months warrants further exploration [46]. These findings reinforce the need for year-round tick prevention strategies, particularly in regions affected by climate change [6,29,58].

The persistence of TBDs in both symptomatic and asymptomatic dogs during the autumn-winter season highlights the ongoing risk posed by these vectors. In the present study, the prevalence of *Babesia* spp. in ticks was similar to that reported in other European countries, where the geographic range of *B*. *canis* has expanded significantly, mainly due to the spread of *D. reticulatus* [25]. These findings underscore the importance of continuous monitoring and preventive measures, even during colder months [42,43].

In the current study, the prevalence of *Babesia* spp. infections varied across dogs of different size, breed, and coat type, but the observed differences were not statistically significant. As expected, the presence of *Babesia* spp. was molecularly confirmed in 29 out of 30 cases (96.7%). Additionally, other TBDs were detected, including *Anaplasma* spp., *Borrelia* spp., and *Mycoplasma* spp., confirming the high risk of co-infections. The cause of death of three dogs was progressive renal and hepatic failure, which ultimately led to multi-organ failure and death of the patients. The presented morphological and biochemical results were performed on the first day, when the diagnosis was made based on the results of the blood smear. The results of laboratory tests did not differ from the results of other patients, however, there was no response to treatment and the clinical condition of the patients deteriorated. In two of these cases, only *Babesia* spp. was detected, and in one case, a mix-infection of *Anaplasma* spp. and *Borrelia* spp.

The relatively low prevalence of *Anaplasma* spp. (3.3%) and *Mycoplasma* spp. (13.33%) aligns with the findings of other studies that have reported lower seroprevalence rates for these pathogens than for *Babesia* spp. in European dogs [59,60]. However, *Borrelia* spp. were identified in 10% of the dogs with babesiosis diagnosis, which is similar to findings from healthy sled and pet dogs from Central and Northern Europe [61]. It also considers other TBPs detected in dogs from this study, including *Anaplasma* spp. and *Mycoplasma* spp. In this study, the prevalence of *Babesia* spp. and *Borrelia* spp. was somewhat higher in large and medium-sized dogs than in small dogs, but these differences were not statistically significant. Similar trends have been reported in other studies (but no linear pattern of risk was observed), suggesting that larger dogs may be more susceptible to TBDs due to increased exposure to tick habitats [62]. In our studies, co-infections were observed in some dogs. Interestingly, co-infections were identified in eight dogs, as shown in Table 3. The most frequent co-infection was *Babesia* spp. and *Mycoplasma* spp., found in five cases. This was followed by co-infections of *Babesia* spp. and *Borrelia* spp. in two dogs, and a single instance of *Anaplasma* spp. paired with *Borrelia* spp. Co-infections may complicate clinical management, which highlights the need for comprehensive diagnostic approaches [14,38]. An example from our study is patient no. 26, which did not survive the treatment despite double administration of imidocarb and the introduction of doxycycline.

Co-infections pose a significant diagnostic challenge in the management of TBDs. In the present study, co-infections were noted in several dogs, and other researchers also reported frequent co-infections in regions where multiple tick species are endemic [7]. Co-infections can exacerbate clinical symptoms and complicate treatment strategies because the presence of multiple pathogens may increase the severity of disease symptoms. For example, dogs co-infected with *B*. *burgdorferi* and *A. phagocytophilum* often present with more severe clinical signs than those infected with a single pathogen [34]. The use of molecular diagnostic techniques, such as PCR, for pathogen detection is an unquestionable strength of this study because these methods offer greater sensitivity and specificity compared to traditional serological tests [63]. However, the variability in clinical signs in infected dogs, ranging from fever and anemia to leukocytosis and thrombocytopenia, highlights the non-specific nature of TBD symptoms. This diagnostic complexity requires a comprehensive approach that considers the possibility of co-infections and employs multiple diagnostic tools to ensure accurate detection and effective treatment [64].

The study also examined ticks collected from dogs, and *I. ricinus* and *D. reticulatus* were identified. The distribution of pathogens among these tick species provides valuable insights into the ecology of TBDs and the dynamics of pathogen transmission. For example, *Borrelia* spp. were detected in 25.6% of *I. ricinus* ticks, which is consistent with other studies, where *I. ricinus* was identified as the main vector for Lyme disease in Europe [36]. The detection of *Babesia* spp. in *D. reticulatus* is also significant because this species is the key vector for *B. canis* [25].

The results of the phylogenetic analysis provide important insights into the genetic diversity and geographic spread of TBDs in Europe. The high similarity between *Babesia canis* sequences identified in this study and those detected in Italy, Turkey, Serbia, Netherlands, Poland, Slovakia, and Russia points to the widespread distribution of this pathogen across Europe [7,22]. It is interesting that the several obtained sequences of *Babesia* spp. were found in the golden jackal. As this is a species migrating northwards, it may be a reservoir of these pathogens. The *A*. *phagocytophilum* clusters with a sequence from Estonia. It is also identical to sequences from more countries, including Norway, Japan, Croatia, Austria, Germany, and Poland, indicating a common genetic lineage across geographic regions [34,35]. These genetic data support the notion that TBDs are not limited by national borders, and that wildlife and domestic animals act as reservoirs and carriers of these pathogens across various regions [65]. The sequences of *M. haemocanis* from our studies are grouped into clades corresponding to samples from countries such as Italy, Turkey, Switzerland, Germany, and others, showing distinct geographic clustering. *Candidatus* M. haematoparvum isolates also demonstrate clear clustering, with notable branches for samples from Spain, Sweden, and Cuba, suggesting some degree of regional specificity. *Borrelia garinii* isolates form distinct clades, including samples from France, Switzerland, Japan, and other countries, indicating a clear regional association. Similarly, *B burgdorferi* clusters include samples from diverse locations such as Russia, the Czech Republic, and the United Kingdom, showing some degree of geographical segregation. The identification of *B. garinii* and *B. burgdorferi* sequences in ticks from Poland further emphasizes the complex epidemiology of Lyme disease in Europe. The presence of these genospecies in ticks collected from both wildlife and domestic animals suggests that these pathogens circulate in a wide range of ecological niches, contributing to sustained transmission of Lyme disease [61,66,67]. The genetic diversity observed in these pathogens underscores the need for ongoing surveillance and molecular studies to monitor the evolution and spread of TBDs across Europe [6,9,30]. The detection of zoonotic pathogens such as *B. burgdorferi* in dogs and ticks also raises public health concerns, particularly regarding the potential for transmission to humans. This underscores the importance of a “One Health” approach that considers the interconnectedness of human, animal, and environmental health in the management of TBDs [5,66].

The present findings have critical implications for both veterinary practice and public health. The persistence of tick activity during the autumn-winter season necessitates a shift in how veterinarians approach TBD prevention and control. Traditional strategies focusing on tick prevention during spring and summer may no longer be sufficient, especially in regions experiencing milder winters due to climate change [7]. Veterinarians should emphasize year-round tick control, incorporating regular screening and the use of acaricides to reduce tick exposure throughout the year [4,37].

Preventive measures are central to the effective management of TBDs. Consistent use of acaricides, environmental control strategies to reduce tick habitats, and public education campaigns are vital components of a comprehensive tick control program [4,37]. Recent advances in the development of vaccines against tick-borne pathogens offer promise in reducing the incidence of these diseases in companion animals [4,17,18]. However, the success of these measures depends on their consistent application and the collaboration of pet owners, veterinarians, and public health officials [4].

Despite the growing body of research, significant knowledge gaps remain. The present findings highlight the need for more research into the seasonal dynamics of tick populations, particularly during winter, to better understand the factors driving year-round tick activity [42,43]. Moreover, the clinical implications of co-infections in dogs require further investigation to develop improved diagnostic and treatment strategies [14,68]. Comprehensive studies are needed to assess the prevalence and impact of co-infections, and to develop broad-spectrum diagnostic tools that can be used year-round [68]. Additionally, increased research is needed to evaluate the prevalence of TBDs at the end of the year, particularly in regions undergoing rapid climatic changes [58]. Addressing these research gaps will be crucial in enhancing our ability to protect companion animals from the persistent threats posed by tick-borne diseases and mitigate their broader impact on public health [5,10].

The present study has certain limitations. Above all, it was conducted on a relatively small group of dogs, which is why statistically significant results were not obtained (*p* > 0.05). However, obtaining a larger research group with symptoms of TBDs in a non-specific period is problematic. The above also applies to ticks. A retrospective study could offer an alternative; however, some of the results could be heterogeneous because veterinarians have different approaches to treatment methods. In addition, the facility does not store biological material that would be critical for PCR tests. The study also examined animals from only one region in Central Europe. It should be noted, however, that this is one of the coldest regions in Poland; therefore, the observed prevalence of TBDs in dogs in the autumn-winter season could be similar to that noted in other regions.

## 5. Conclusions

This study contributes valuable insights into the prevalence and clinical manifestations of TBDs in dogs during the autumn-winter season in Poland. The results underscore the importance of year-round vigilance in tick prevention and control, driven by the shifting dynamics of tick populations and the persistent risk of pathogen transmission. The confirmation of *Babesia* spp., co-infections, and the genetic diversity of detected pathogens all highlight the complex and evolving nature of TBDs in companion animals. By adopting a proactive approach involving continuous monitoring, broad-spectrum diagnostics, and integrated prevention strategies, veterinary practitioners can mitigate the impact of these diseases on canine health. As climate change continues to alter tick ecology, ongoing research and collaboration will be essential in addressing the emerging challenges associated with TBDs in both animals and humans.

## Figures and Tables

**Table 1:** Primers and PCR conditions.

Species	Target Gene	Name of Primer	Primer Sequences (5′-3′)	Expected Size (bp)	PCR Cycle Conditions	Reference
*Anaplasma* spp./ *Ehrlichia* spp.	16S rRNA	EHR16SD EHR16SR	GGT ACC TAC AGA AGA AGT CC TAG CAC TCA TCG TTT ACA GC	345	94 °C/5 min; 40 cycles: 94 °C/30 s, 52 °C/45 s, 68 °C/45 s; 68 °C/5 min	[52]
*Babesia* spp.	18 rRNA	BTH 18S 1stF BTH 18S 1stR BTH 18S 2ndF BTH 18S 2ndR	GTG AAA CTG CGA ATG GCT CAT TAC AAG TGA TAA GGT TCA CAA AAC TTC CC GGC TCA TTA CAA CAG TTA TAG TTT ATT TG CGG TCC GAA TAA TTC ACC GGA T	1400–1600	94 °C/10 min; 30 cycles: 94 °C/30 s, 55 °C/30 s, 68 °C/1.5 min; 68 °C/1 min 94 °C/10 min; 35 cycles: 94 °C/30 s, 55 °C/30 s, 68 °C/1.5 min; 68 °C/1 min	[53]
*Borrelia* sensu lato	5S-23S rRNA	Bb23S3 Bb23Sa Bb23S3nF Bb23S3anR	CGA CCT TCT TCG CCT TAA AGC TAA GCT GAC TAA TAC TAA TTA CCC CTG CGA GTT CGC GGG AGA TCC TAG GCA TTC ACC ATA	226–266	94 °C/5 min; 35 cycles: 94 °C/30 s, 55 °C/30 s, 68 °C/40 s; 68 °C/5 min 94 °C/5 min; 35 cycles: 94 °C/30 s, 59 °C/30 s, 68 °C/40 s; 68 °C/5 min	[54]
*Mycoplasma* spp.	16S rRNA	Mycoplasma 16SrRNA F2 Mycoplasma 16SrRNA R2	ATA CGG CCC ATA TTC CTA CG TGC TCC ACC ACT TGT TCA	595	94 °C/10 min; 40 cycles: 95 °C/30 s, 60 °C/30 s, 72 °C/30 s; 72 °C/10 min	[55]

**Table 2:** Results of clinical examination and blood analysis.

Parameter	Results
Breed	purebred 11 dogs (37%) mixed 19 dogs (63%)
Sex	males 13 (43%) females 17 (57%).
Age	≤1-year-old 5 dogs (16.7%) 2–7 years-old 20 dogs (66.7%) ≥8-years-old 5 dogs (16.7%)
Size	≤5 kg 5 dogs (16.7%) 5–25 kg 14 dogs (46.7%) ≥25 kg 11 dogs (36.7%)
Coat	short coat 11 dogs (37%) long coat 16 dogs (53%) wire coat 3 dogs (10%)
Protection against ectoparasites	yes 6 dogs (20%) no 24 dogs (80%)
Temperature	>39.0 °C 22 dogs (73.3%); m = 39.75 °C, SD = 0.58, SE = 0.12 ≤39.0 °C 8 dogs (26.7%); m = 38.45 °C, SD = 0.47, SE = 0.17
RBC 5.65–8.87 M/µL	↓ 17 dogs (56.7%); m = 4.04 M/µL, SD = 1.33, SE = 0.32 = 13 dogs (43.3%); m = 6.55 M/µL, SD = 0.79, SE = 0.22
PLT 148–484 K/µL	↓ 24 dogs (80%); m = 75.33 K/µL, SD = 33.77, SE = 6.89 = 6 dogs (20%); m = 162.83 K/µL, SD = 50.21, SE = 20.49
WBC 5.05–16.76 K/µL	↑ 9 dogs (30%); m = 20.07 K/µL, SD = 1.87, SE = 0.62 ↓ 8 dogs (26.7%); m = 4.11 K/µL, SD = 0.71, SE = 0.25 = 13 dogs (43.3%); m = 9.65 K/µL, SD = 3.32, SE = 0.92
Urine color	dark urine 16 dogs (53.3%) normal urine 14 dogs (46.7%)
ALKP 23–212 U/L	↑ 7 dogs (23.3%); m = 1078.43 U/I, SD = 478.41SE = 180.78 = 23 dogs (76.7%); m = 180.91 U/I, SD = 264.68, SE = 55.19
ALT 10–25 U/L	↑ 6 dogs (20%); m = 603.67 U/I, SD = 453.97, SE = 185.34 = 24 dogs (80%); m = 96.27 U/I, SD = 56.47, SE = 11.53
AST 0–50 U/L	↑ 19 dogs (63.3%); m = 184.36 U/I (SD = 207.91, SE = 47.71 = 11 dogs (36.7%); m = 33.82 U/I, SD = 14.77, SE = 4.45
BUN 7–27 mg/dL	↑ 10 dogs (33.3%); m = 192.67 mg/dL, SD = 155.04, SE = 53.01 = 20 dogs (66.7%); m = 36.85 mg/dL, SD = 36.02, SE = 8.06
CREA 0.5–1.8 mg/dL	↑ 9 dogs (30%); m = 111.36 mg/dL, SD = 184.34, SE = 61.45 = 21 dogs (70%); m = 1.53 mg/dL, SD = 0.64, SE = 0.14
TBIL 0–0.9 mg/dL	↑ 4 dogs (13.3%); m = 2.8 mg/dL, SD = 1.26, SE = 0.63 = 26 dogs (86.7%); m = 0.41 mg/dL, SD = 0.24, SE = 0.05

Legend: RBC—red blood cell; PLT—platelet count; WBC—white blood cell; ALKP—alkaline phosphatase; ALT—alanine aminotransferase; AST—aspartate aminotransferase; BUN—blood urea nitrogen; CREA—creatinine; TBIL—total bilirubin; =—normal level; ↓—decreased level; ↑—increased level; m—mean values; SD—standard deviation; SE—standard error.

**Table 3:** Polymerase chain reaction results in dogs, with characteristics of dogs.

No.	*Breed*	*Sex*	*Age*	*Coat*	*Size*	*Babesia* spp.	*Anaplasma* spp.	*Borrelia* spp.	*Mycoplasma* spp.
1	mixed	male	5	short coat	small	**+**	**−**	**−**	**−**
2	purebred	female	9	long coat	medium	**+**	**−**	**−**	**−**
3	mixed	male	3	short coat	large	**+**	**−**	**−**	**−**
4	mixed	female	4	short coat	large	**+**	**−**	**−**	**+**
5	mixed	female	8	long coat	small	**+**	**−**	**−**	**−**
6	purebred	male	2	long coat	medium	**+**	**−**	**+**	**−**
7	mixed	female	3	long coat	large	**+**	**−**	**−**	**−**
8 **†**	mixed	male	1	short coat	medium	**+**	**−**	**−**	**−**
9	mixed	female	4	long coat	large	**+**	**−**	**−**	**+**
10	purebred	female	1	long coat	large	**+**	**−**	**−**	**−**
11	purebred	male	4	short coat	medium	**+**	**−**	**−**	**−**
12	purebred	male	3	wire coat	large	**+**	**−**	**+**	**−**
13	mixed	male	6	long coat	small	**+**	**−**	**−**	**−**
14	mixed	female	1	long coat	medium	**+**	**−**	**−**	**−**
15	purebred	female	1	short coat	large	**+**	**−**	**−**	**−**
16	purebred	male	8	short coat	medium	**+**	**−**	**−**	**−**
17	purebred	female	7	wire coat	medium	**+**	**−**	**−**	**−**
18	mixed	male	4	long coat	small	**+**	**−**	**−**	**−**
19	mixed	male	7	long coat	large	**+**	**−**	**−**	**+**
20 **†**	mixed	female	1	short coat	medium	**+**	**−**	**−**	**−**
21	mixed	male	6	long coat	medium	**+**	**−**	**−**	**−**
22	mixed	male	4	short coat	medium	**+**	**−**	**−**	**−**
23	mixed	female	7	long coat	small	**+**	**−**	**−**	**−**
24	purebred	male	8	short coat	medium	**+**	**−**	**−**	**−**
25	mixed	female	2	short coat	medium	**+**	**−**	**−**	**+**
26 **†**	mixed	female	6	long coat	large	**−**	**+**	**+**	**−**
27	purebred	female	5	long coat	medium	**+**	**−**	**−**	**−**
28	mixed	female	5	long coat	large	**+**	**−**	**−**	**−**
29	mixed	male	8	long coat	large	**+**	**−**	**−**	**+**
30	purebred	female	2	short coat	medium	**+**	**−**	**−**	**−**

Legend: †—dogs that died.

**Table 4:** Occurrence of pathogens in tick species by sex, engorgement status, and pathogen type during the autumn-winter season.

Pathogen		*Ixodes ricinus* (39/45; 86.7%)					*Dermacentor reticulatus* (6/45; 13.3%)				
	Total	Male		Female		Subtotal	Male		Female		Subtotal
	*n* = 45	21/39; 53.8%		18/39; 46.2%;		*n* = 39	2/6; 33.3%		4/6; 66.7% 95%		*n* = 6
		ne (*n* = 7)	e (*n* = 0)	ne (*n* = 10)	e (*n* = 3)		ne (*n* = 0)	e (*n* = 0)	ne (*n* = 1)	e (*n* = 1)	
*Babesia* spp.	3/45; 6.7% 95% CI: 0–10.6%	1/7; 14.3% 95% CI: 0–40.2%	0	1/10; 10.0% 95% CI: 0.1–25.6%	1/3; 33.3% 95% CI: 0–86.7%	3/39; 7.7% 95% CI: 0.3–12.9%	0	0	0	0	0
*Mycoplasma* spp.	3/45; 6.7% 95% CI: 0–10.6%	1/7; 14.3% 95% CI: 0–40.2%	0	2/10; 20% 95% CI: 2.3–47.7%	0	3/39; 7.7% 95% CI: 0.3–12.9%	0	0	0	0	0
*Borrelia* spp.	11/45; 24.4% 95% CI: 14.4–36.6%	4/7; 57.1% 95% CI: 20.5–93.8%	0	4/10; 40% 95% CI: 13.3–66.7%	2/3; 66.7% 95% CI: 13.3–100%	10/39; 25.6% 95% CI: 14.3–37.7%	0	0	0	1/1; 100%	1/6; 16.7% 95% CI: 0–46.5%
*Anaplasma* spp.	5/45; 11.1% 95% CI: 2.9–17.1%	1/7; 14.3% 95% CI: 0–40.2%	0	3/10; 30% 95% CI: 3.2–46.8%	0	4/39; 10.3% 95% CI: 2.6–15.4%	0	0	1/1; 100%	0	1/6; 16.7% 95% CI: 0–46.5%

Legend: ne—non-engorged; e—engorged.

## Data Availability

The original contributions presented in the study are included in the article. Further inquiries can be directed to the corresponding author.

## Supplementary Materials

The following supporting information can be downloaded at: https://www.mdpi.com/article/10.3390/pathogens13121132/s1. Supplementary Material S1: Confidence intervals (95% CI) for clinical and epidemiological parameters in dogs diagnosed with tick-borne diseases during the autumn-winter season in Poland.
