# Supplementary material for: Clinical Cases of Tick-Borne Diseases in Dogs During the Autumn-Winter Season in Poland
Source: Pathogens. 2024 Dec 21;13(12):1132. doi: 10.3390/pathogens13121132 (PMC11678369; doi:10.3390/pathogens13121132)
Supplement: Supplementary file 1 [file pathogens-13-01132-s001.zip › pathogens-3295913-supplementary.pdf]

Supplementary Material S1. Confidence intervals (95% CI) for clinical and epidemiological parameters in dogs diagnosed with tick-borne diseases during the autumn-winter season in Poland .

| Clinical parameters | | | |
|---|---|---|---|
| Parameter | Mean | Lower CI | Upper CI |
| Elevated Body Temperature | 39.75 | 39.51 | 39.99 |
| Normal Body Temperature | 38.45 | 38.12 | 38.78 |
| Anemic RBC Levels | 4.04 | 3.41 | 4.67 |
| Normal RBC Levels | 6.55 | 6.12 | 6.98 |
| Leukocytosis | 20.07 | 18.85 | 21.29 |
| Leukopenia | 4.11 | 3.62 | 4.60 |
| Normal Leukocyte Count | 9.65 | 7.85 | 11.45 |
| Thrombocytopenia Platelet Count | 75.33 | 61.82 | 88.84 |
| Normal Platelet Count | 162.83 | 122.65 | 203.01 |
| Normal Creatinine Levels | 1.53 | 1.26 | 1.80 |
| Elevated Creatinine Levels | 111.36 | -9.07 | 231.79 |
| Normal BUN Levels | 36.85 | 21.06 | 52.64 |
| Pathological BUN Levels | 192.67 | 96.58 | 288.76 |
| Elevated AST Levels | 184.36 | 90.87 | 277.85 |
| Normal AST Levels | 33.82 | 25.09 | 42.55 |
| Pathological ALT Levels | 603.67 | 240.43 | 966.91 |
| Normal ALT Levels | 96.27 | 73.68 | 118.86 |
| Elevated ALKP Levels | 1078.43 | 724.03 | 1432.83 |
| Normal ALKP Levels | 180.91 | 72.74 | 289.08 |
| Elevated Bilirubin Levels | 2.80 | 1.57 | 4.03 |
| Normal Bilirubin Levels | 0.41 | 0.32 | 0.50 |
| **Epidemiological parameters (dogs)** | | | |
| Parameter | % | Lower CI | Upper CI |
| Purebred dogs | 37.0 | 19.4 | 54.0 |
| Mixed breeds | 63.0 | 46.1 | 80.6 |
| Male dogs | 43.0 | 25.6 | 61.1 |

| | | | |
|---|---|---|---|
| Female dogs | 57.0 | 39.0 | 74.4 |
| ≤1-year-old | 16.7 | 3.3 | 30.0 |
| 2-7 years | 66.7 | 49.8 | 83.5 |
| ≥8-years-old | 16.7 | 3.3 | 30.0 |
| Small breeds | 17.0 | 3.3 | 30.0 |
| Medium breeds | 53.0 | 35.5 | 71.2 |
| Large breeds | 30.0 | 13.6 | 46.4 |
| Short-coated | 37.0 | 19.4 | 54.0 |
| Long-coated | 53.0 | 35.5 | 71.2 |
| Wire-coated | 10.0 | 0.0 | 20.7 |
| Protected against ectoparasites | 20.0 | 5.7 | 34.3 |
| Not protected against ectoparasites | 80.0 | 65.7 | 94.3 |
| Babesiosis confirmed by PCR | 96.7 | 86.1 | 100.0 |
| Babesiosis not confirmed by PCR | 3.3 | 0.0 | 13.9 |
| Mixed-breed dogs positive for *Anaplasma* spp. | 5.3 | 0.0 | 12.5 |
| Large dogs positive for *Babesia* spp. | 36.7 | 19.4 | 54.0 |
| Medium dogs positive for *Babesia* spp. | 46.7 | 28.8 | 64.5 |
| Small dogs positive for *Babesia* spp. | 16.7 | 3.3 | 30.0 |
| Medium/large dogs positive for *Borrelia* spp. | 10.0 | 0.0 | 20.7 |
